# *Haemaphysalis longicornis* Ticks as Reservoir and Vector of Severe Fever with Thrombocytopenia Syndrome Virus in China

**DOI:** 10.3201/eid2110.150126

**Published:** 2015-10

**Authors:** Li-Mei Luo, Li Zhao, Hong-Ling Wen, Zhen-Tang Zhang, Jian-Wei Liu, Li-Zhu Fang, Zai-Feng Xue, Dong-Qiang Ma, Xiao-Shuang Zhang, Shu-Jun Ding, Xiao-Ying Lei, Xue-jie Yu

**Affiliations:** Shandong University, Jinan, China (L.-M. Luo, L. Zhao, H.-L. Wen, J.-W. Liu, L.-Z. Fang, X.-S. Zhang, X.-Y. Lei, X.-j. Yu);; Huangdao District Center for Disease Control and Prevention, Qingdao City, China (Z.-T. Zhang, Z.-F. Xue, D.-Q. Ma);; Shandong Province Center for Disease Control and Prevention, Jinan (S.-J. Ding);; University of Texas Medical Branch, Galveston, Texas, USA (X.-j. Yu)

**Keywords:** bunyavirus, phlebovirus, severe fever with thrombocytopenia syndrome, severe fever with thrombocytopenia syndrome virus, SFTSV, ticks, Haemaphysalis longicornis, viruses, vector-borne infections, transmission, transstadial transmission, transovarial transmission, tickborne transmission, vector, host, larvae, nymphs, adult ticks, China

## Abstract

Severe fever with thrombocytopenia syndrome (SFTS) is an emerging hemorrhagic fever in East Asia caused by SFTS virus (SFTSV), a newly discovered phlebovirus. The *Haemaphysalis longicornis* tick has been suspected to be the vector of SFTSV. To determine whether SFTSV can be transmitted among ticks, from ticks to animals, and from animals to ticks, we conducted transmission studies between developmental stages of *H. longicornis* ticks and between ticks and mice. Using reverse transcription PCR, we also analyzed the prevalence of SFTSV infection among *H. longicornis* ticks collected from vegetation in Shandong Province, China. Our results showed a low prevalence of SFTSV among collected ticks (0.2%, 8/3,300 ticks), and we showed that ticks fed on SFTSV-infected mice could acquire the virus and transstadially and transovarially transmit it to other developmental stages of ticks. Furthermore, SFTSV-infected ticks could transmit the virus to mice during feeding. Our findings indicate ticks could serve as a vector and reservoir of SFTSV.

## Abstract 2

Transstadial and transovarial virus transmission occur among ticks, and transmission to mice can occur through a tick bite.

Severe fever with thrombocytopenia syndrome (SFTS) is an emerging hemorrhagic fever caused by SFTS virus (SFTSV), a newly discovered phlebovirus in the family *Bunyaviridae* (*1*,*2*). SFTS was reported in China in 2009 (*1*) and subsequently in Korea and Japan (*3*,*4*). Approximately 1,000 SFTS cases are reported each year in China (*5*), where the case-fatality rate is 6.3%–12.0% (*1*,*5*). SFTS cases occur in rural areas of China, where there are shrubs, grasslands, or both, and a high density of *Haemaphysalis longicornis* ticks (*1*,*6*–*9*). Since the first discovery of SFTSV, transmission of the virus by ticks, especially the *H. longicornis* tick, has been proposed (*1*). However, it has not been determined whether SFTSV can be transmitted transstadially and transovarially in ticks or whether ticks can transmit SFTSV to animals. To determine the possibility of such transmission and to determine whether ticks might be a reservoir for SFTSV, we investigated ticks as a possible vector by determining tickborne transmission of SFTSV during the tick developmental stages and transmission of SFTSV to animals.

## Materials and Methods

### Collection of Ticks from Vegetation

During June and July 2013, we collected ticks (questing larvae, nymphs, and adults) in Jiaonan County, Shandong Province, China (119°30′–120°30′, 35°35′–36°08′), by flagging over vegetation with a 1-m^2^ flannel flag. Tick collection was performed for 4 days during the first week of each month between 10:00 am and 12:00 pm and between 2:00 pm and 5:00 pm; different sites were used for each collection. The ticks were frozen at −80°C until use. Tick species and developmental stages were identified morphologically, and the tick species was molecularly confirmed. The use of the animals and the collection of samples were approved by the bioethics committee of School of Public Health, Shandong University.

### Tick Nucleotide Extraction and Identification by PCR

Ticks were grouped according to their developmental stages; each group contained 40 larvae, 20 nymphs, or 5 adult ticks. Sex of the ticks was ignored. Ticks in each group were homogenized by using metal beads and Buffer RLT in a TissueLyser LT (both from QIAGEN, Hilden, Germany). The total nucleotides (DNA and RNA) were extracted simultaneously by using the AllPrep DNA/RNA Mini Kit (QIAGEN) according to the manufacturer’s instructions. Two larval ticks, 1 nymphal tick, and 2 adult ticks were processed individually for rRNA extraction and PCR amplification of the tick mitochondrial 16S rRNA gene. The tick mitochondrial 16S RNA gene forward primer (5′-AGTATTTTGACTATACAAAGGTATTG-3′) and reverse primer (5′-GTAGGATTTTAAAAGTTGAACAAACTT-3′) were designed in this study by using a previously published sequence (GenBank accession no. KC203361) as template. The PCR cycle consisted of an initial DNA denaturation step at 95°C for 2 min; 35 cycles of 30 s at 92°C, 30 s at 55°C, and 45 s at 72°C; and a final extension step of 10 min at 72°C. The PCR product was 402 bp and was sequenced on both strands.

### Detection of SFTSV in Ticks by Reverse Transcription PCR (RT-PCR)

The extracted SFTSV RNA was amplified by using a one-step RT-PCR (Access RT-PCR System Kit, Promega, Madison, WI, USA) with primers derived from the small RNA segment of the virus. The RT-PCR primers (CAGCCAGTTTACCCGAACAT and GAAAGACGCAAAGGAGT) and PCR protocol were described previously (*10*). The one-step RT-PCR consisted of 45 min at 45°C and 2 min at 94°C; 40 cycles of 30 s at 94°C, 1 min at 60°C, and 2 min at 68°C; and a final extension of 10 min at 68°C. The RT-PCR product was used as template for a nested PCR, which consisted of a denaturing cycle of 5 min at 95°C; 30 cycles of 20 s at 94°C, 30 s at 56°C, and 1 min at 72°C; and a final extension of 10 min at 72°C. A negative control with sterilized distilled water was run simultaneously. Nested PCR was performed with primers (5′-TGGCTCCGCGCATCTTCACA-3′ and 5′-AGAGTGGTCCAGGATTGCTGTGG-3′), using the RT-PCR product as template (*11*). The amplified DNA was subjected to electrophoresis on a 0.8% agarose gel and visualized under ultraviolet light. The desired 560-bp DNA band was excised and purified from the gel by using the Gel Extraction Kit (QIAGEN). The purified PCR product was cloned into a pMD 19-T vector (TaKaRa Bio Inc., Shiga, Japan), according to the manufacturer’s instructions. Positive clones were sequenced on both strands.

### Phylogenetic Analysis

The SFTSV sequences and tick mitochondrial 16S rRNA gene sequences derived from tick nucleotides were compared with sequences in GenBank by using BLAST (http://blast.ncbi.nlm.nih.gov/Blast.cgi). Phylogenetic trees were constructed by using the neighbor-joining method in MEGA5 (*12*), and the robustness of the trees was tested by using 1,000 bootstrap replications. The sequences generated in this study were deposited in GenBank (accession nos. KP300821, KR259990–KR25993 for ticks and KP197680–KP197687 for SFTSV).

### Establishment of a Tick Colony and Transmission Feeding Experiments

A colony of parthenogenic *H. longicornis* tick was established in our laboratory. Initially, adult female ticks collected from vegetation were placed inside a bag attached to a rabbit ear to feed. Each engorged tick was allowed to lay eggs in a plastic tube. Approximately a half clutch of eggs from each female tick was used for RNA extraction and testing for SFTSV by RT-PCR. Larvae were allowed to hatch from the remaining half clutches, and larvae from a single tick that was negative for SFTSV by RT-PCR were used for all subsequent experiments.

SFTSV acquisition and transmission feeding were performed for all stages of ticks by using Kunming mice (Shandong University Experimental Animal Center, Jinan City, China). For the experiments, ticks were placed in feeding capsules, which were prepared from the top of 1-mL screwcap cryovials. The top part of the tube beneath the cap was cut, and the cap of the tube was punctured by using a 26-gauge needle for adequate air exchange. Mice were anesthetized by intraperitoneal injection of 0.1 mL of 10% chloral hydrate. Hair on the back of each mouse was trimmed close to the skin by using a small electric razor, and a capsule was affixed to the back with Kamar glue (Kamar Products Inc., Zionsville, IN, USA). To secure the capsule in place, a round patch of cloth (≈1 cm diameter) with a hole in the middle that was slightly smaller than the capsule (to allow access to the capsule) was placed over the capsule and affixed to the skin with glue. A collar made from thin plastic was placed around the body of the mouse in front of the capsule to prevent the mouse from removing the capsule during grooming.

A total of 50 larvae, 15 nymphs, or 1 adult tick was placed in the capsule on each mouse 24 h after the capsule was affixed to the mouse. Ticks in the capsule were observed daily and collected after completion of feeding. For the virus acquisition feeding, each mouse was inoculated intraperitoneally with DH82 cells containing 10^6^ plaque forming units of SFTSV, and ticks were fed on the SFTSV-infected mice for 3–8 days until engorged. For virus transmission feeding, SFTSV-infected ticks were fed on noninfected mice for 3–8 days until engorged. We collected blood samples from each mouse 7, 14, and 21 days after tick feeding for detection of SFTSV. The SFTSV in the laboratory-reared ticks and in the mouse blood was detected by RT-PCR using the primer pair CAGCCACTTCACCCGAACAT and AAGGAAAGACGCAAAGGAG, which was designed in this study. The amplification cycles were the same as those described above for the RT-PCR for detection of SFTSV in ticks from vegetation. The PCR product was 560 bp.

### Detection of SFTSV RNA and Antibody in Blood of Mice after Tick Feeding

Mouse blood samples were collected weekly for 3 weeks. Total RNA was extracted from each blood sample by using the RNeasy Mini Kit (QIAGEN) and was used as a template for amplification (Access RT-PCR System; Promega) of SFTSV. Primers for RT-PCR and the PCR protocol were as described in the preceding paragraph. SFTSV antibody was assayed by using SFTSV-infected DH82 cells as antigens. The cells were cultivated in a 96-well plate, fixed with 4% paraformaldehyde, and used as antigens for immunofluorescence assays (IFAs) to detect SFTSV antibodies in serum samples from mice fed on by SFTSV-infected ticks.

## Results

### The Prevalence of SFTSV in Ticks from Vegetation

**Figure 1:**
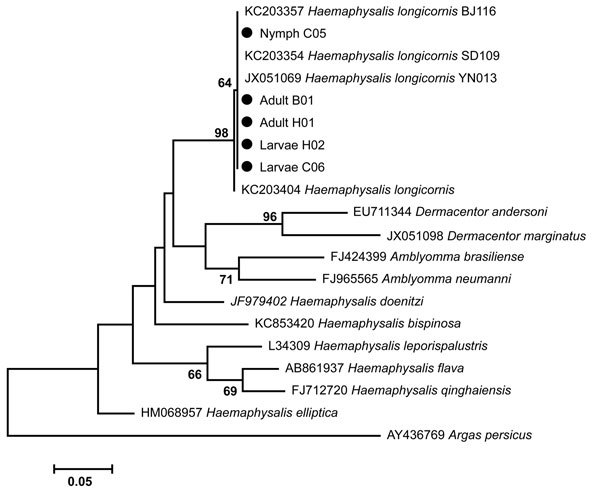
Phylogenetic analysis of mitochondrial 16S rRNA gene of ticks collected during June–July 2014 from Jiaonan County, Shandong Province, China. The results showed that the larval, nymphal, and adult ticks (indicated by black dots) were all *Haemaphysalis longicornis* ticks. Scale bar represents nucleotide substitutions per site.

**Figure 2:**
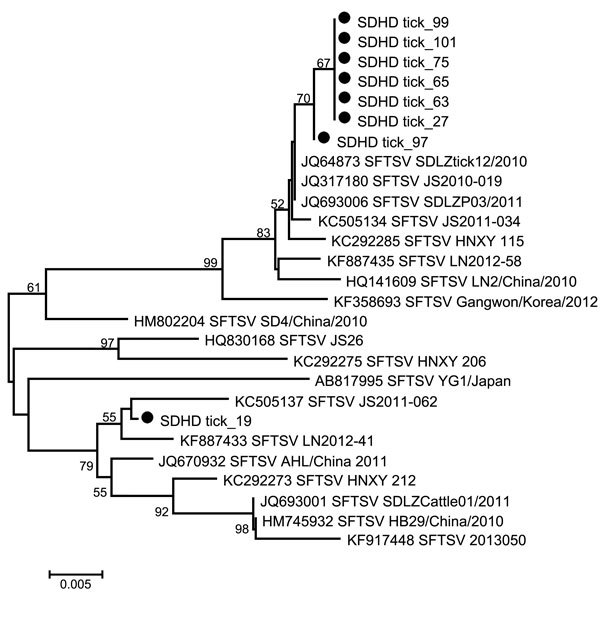
Phylogenetic analysis of severe fever with thrombocytopenia syndrome virus (SFTSV) small segment sequences from ticks collected during June–July 2014 from Jiaonan County, Shandong Province, China. Dots indicate SFTSV sequences amplified from ticks in this study; GenBank accession numbers are shown for other sequences. Scale bar represents nucleotide substitutions per site.

We collected 3,300 ticks from vegetation, morphologically identified them as *H. longicornis* ticks, and molecularly confirmed the identification by sequencing the mitochondrial 16S RNA gene of representatives of larval, nymphal, and adult ticks (Figure 1). To detect SFTSV, we pooled the ticks according to their developmental stage, and tested each pool for SFTSV RNA by RT-PCR and nested PCR. The prevalence of SFTSV infection in each stage of the tick was determined by the assumption that a positive pool of ticks contained 1 SFTSV-infected tick. The prevalence of SFTSV infection was 0.2% (8/3,300) among ticks of all stages. The prevalence of SFTSV infection was 0 among 120 larvae, 0.1% (2/1,620) among nymphs, and 0.4% (6/1,560) among adult ticks. Phylogenetic analysis showed that all sequences of SFTSV amplified from ticks were clustered with SFTSV from Shandong Province and other places in China (Figure 2). These results suggested that nymph and adult ticks were infected with SFTSV, but the rate of carriage was very low.

### SFTSV Infection of Ticks by Acquisition Feeding and Tick Transovarial Transmission of SFTSV

To determine whether ticks could be infected by SFTSV, we fed all stages (larvae, nymphs, and adults) of *H. longicornis* ticks on SFTSV-infected mice. In larval acquisition feeding, the engorged larvae were tested for SFTSV by RT-PCR before or after molting. Five larval ticks were grouped together as a pool for RNA extraction and RT-PCR amplification of SFTSV. The minimum infection rate (assuming that an infected pool of ticks contained 1 SFTSV-infected tick) was 18% (9/50) for engorged larval pools and 11.7% (14/120) for molted nymphs (Table 1). These results indicated that larvae could acquire SFTSV from infected mice and transstadially transmit SFTSV to nymphs.

**Table 1:** Rate of SFTSV infection among *Haemaphysalis longicornis* ticks after virus acquisition feeding and molting*

Tick stage	No. SFTSV-infected ticks after acquisition feeding /no. total (%)	No. ticks with transstadially or transovarially transmitted SFTSV/no. total (%)
Larval	9/50 (18.0)	14/20 (70.0)
Nymphal	10/10 (100)	2/10 (20.0)
Adult	6/13 (46.2)	7/13 (53.8)
*SFTSV, severe fever with thrombocytopenia syndrome virus.		

In nymphal acquisition feeding, 10 engorged nymphs were tested individually, and all were found to be infected with SFTSV; however, only 20% (2/10) of the adults ticks derived from the nymphs were infected with SFTSV (Table 1). These results indicated that nymphs could acquire SFTSV from infected mice and transstadially transmit SFTSV to adult ticks.

For adult tick acquisition feeding, uninfected adult ticks were fed on SFTSV-infected mice until completion of feeding. Thirteen engorged adult ticks were tested individually for SFTSV, and 6 (46.2%) of them were infected with SFTSV, as determined by RT-PCR. Thirteen engorged adult ticks had oviposited, and the larvae hatched. Larvae derived from each tick were tested as a group for SFTSV RNA by RT-PCR, and 53.8% (7/13) of the larval pools were infected with SFTSV (Table 1). The results of these experiments indicated that adult female ticks could transovarially transmit SFTSV.

### Tick Transmission of SFTSV to Mice

To determine whether SFTSV could be transmitted to mice by tick bite, we fed SFTSV-infected nymphs and adults on Kunming albino mice. For study of nymph feeding transmission of SFTSV, 10 uninfected mice were fed upon by 10 nymphs molted from larvae that fed on SFTSV-infected mice until engorged. At 7, 14, and 21 days after the feeding, blood samples were obtained from the mice and examined by RT-PCR to detect SFTSV; viral RNA was detected in 40% (4/10) of the mice. These results indicated that nymphs transmitted SFTSV to mice through feeding.

For study of adult tick feeding transmission of SFTSV, the adult ticks were molted from engorged nymphs obtained from the 10 mice mentioned above. To determine whether SFTSV was transmitted from nymphs to adult ticks and whether adult ticks could transmit SFTSV to mice, 20 adult ticks were fed individually on 20 naive mice until engorged. At 7, 14, and 21 days after the feeding, blood samples were obtained from the mice and examined by RT-PCR to detect SFTSV; viral RNA was detected in 10.0% (2/20) of the mice on days 7 and 14. These results indicated that the adult ticks used in this study were infected in the larval stage by feeding on SFTSV-infected mice.

**Figure 3:**
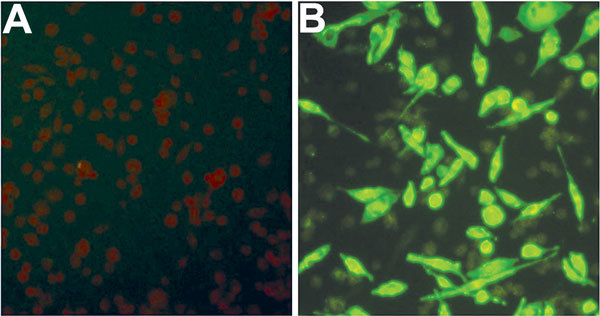
Immunofluorescence assay detection of severe fever with thrombocytopenia syndrome virus (SFTSV) antibodies in serum samples from mice fed by SFTSV-infected ticks. A) Normal mouse serum (negative control) reacting with SFTSV-infected DH82 cells; B) infected mouse serum (1:128, mouse no. 4) reacting with SFTSV-infected DH82 cells.

Using an IFA, we determined whether SFTSV antibodies were present in serum samples from mice fed upon by nymphal and adult ticks. All SFTSV RT-PCR–positive mouse serum samples were also positive by IFA at various titers (Table 2; Figure 3), but none of the SFTSV RT-PCR–negative mouse samples were positive by IFA. The results indicated that larvae acquired SFTSV from infected mice and transmitted the virus transstadially to nymphs and adult ticks, and nymphs and adult ticks transmitted SFTSV to mice during feeding.

**Table 2:** Detection of SFTSV RNA and antibodies in serum samples from mice fed on by SFTSV-infected *Haemaphysalis longicornis* ticks*

Mouse no.	IFA antibody titer	RT-PCR detection of RNA
Fed on by nymphal ticks		
1	128	Positive
2	1,024	Positive
3	64	Positive
4	512	Positive
Fed on by adult ticks		
5	64	Positive
6	128	Positive

*IFA, indirect immunofluorescence assay; RT-PCR, reverse transcription PCR; SFTSV, severe fever with thrombocytopenia syndrome virus.

## Discussion

SFTSV has been believed to be a tick-borne virus since it was first discovered because it was detected in ticks collected from animals (*1*). However, detection of a virus in feeding ticks collected from animals does not confirm that the tick is a vector or reservoir of the virus; it is possible that ticks can acquire a virus from the blood of an infected animal but not maintain it transstadially and transmit it during feeding. Several studies have tried to demonstrate that ticks are a vector and reservoir of SFTSV (*13*,*14*). These studies either failed to detect SFTSV in a large quantity of ticks (n = 11,739) (*13*) or demonstrated very low prevalence of SFTSV in ticks collected from vegetation (*14*). A recent study from South Korea demonstrated that SFTSV was detected in all developmental stages of *H. longicornis* ticks, but the prevalence of infection was low in larvae (0.6% [2/350]) and nymphs (0.4% [38/10, 436]) (*14*). In this study, we demonstrated that the prevalence of SFTSV infection in ticks collected from vegetation is also low (0.2%). A recent study reported a slightly higher prevalence of SFTSV infection (2.2%) in *H. longicornis* ticks collected from vegetation from Shandong Province (*15*). The findings from these studies demonstrate that the prevalence of SFTSV infection in tick populations is very low. The reason for the low SFTSV infection rate in nature is not clear. It may be that SFTSV is detrimental to the infected tick and affects its survival. The low SFTSV prevalence in ticks suggests that ticks alone may not be sufficient to maintain SFTSV in nature; circulation of SFTSV between the tick vector and mammalian amplifying hosts may be required for SFTSV maintenance in nature. In previous studies, we and others demonstrated that SFTSV can infect domesticated animals (goats, sheep, cattle, dogs, and chickens) (*6*–*9*) and wild small mammals (mice, rats, and Asian house shrews) (*16*). These animals may be amplifying hosts and ticks may be a vector and reservoir host of SFTSV.

This study and previous studies (*15*,*17*) demonstrated that *H. longicornis* is the predominant tick species in eastern China. *H. longicornis* ticks feed on domesticated and wild animals, including goats, sheep, cattle, pigs, deer, cats, dogs, rats, mice, hedgehogs, chickens, and other birds, as well as on humans (*18*). In rural China, domesticated animals, especially goats and dogs, often roam freely in the environment and maintain a high tick population around farm houses and may increase the risk of SFTSV infection in humans.

One limitation of this study is that a single species of tick was evaluated as a vector and reservoir of SFTSV. The virus has also been detected in *Rhipicephalus microplus* (formerly *Boophilus microplus*), *Amblyomma testudinarium*, and *Ixodes nipponensis* ticks in China and South Korea (*19*,*20*). However, these ticks are not dominant ticks in eastern China, and whether these tick species play a role in the natural cycle of SFTSV needs to be further investigated. SFTSV has also been detected by RT-PCR in *Leptotrombidium scutellare* mites collected from *Apodemus agrarius* mice and from *Laelaps echidnina* mites collected from *A. agrarius* mice and goats in Jiangsu Province, China (*21*), and from the gadfly (species not defined) in the family *Tabanidae* in Henan Province, China (*17*). Other than ticks, the role of other bloodsucking insects as the vector and reservoir for SFTSV needs to be further investigated.

In this study, we demonstrated that *H. longicorn* ticks at each developmental stage acquired SFTSV during feeding on experimentally infected mice and transmitted SFTSV to mice during feeding. For acquisition feeding, the larval tick appeared to be less efficient than nymphal and adult ticks at acquiring SFTSV from infected mice. However, the difference between infection acquisition for larval ticks and for nymphal and adult ticks may be due to the manner in which we determined the prevalence of SFTSV infection among the different stages of ticks. For larval ticks, prevalence was determined on the basis of the minimum infection rate that was calculated by using pools of ticks; for nymphal and adult ticks, prevalence was determined on the basis of the infection rate that was calculated by using individual ticks. The minimum infection rate may underestimate the true infection rate because a positive pool is counted for only 1 positive tick even though the pool may contain >1 positive tick. We also demonstrated that SFTSV can be transstadially transmitted from larvae to nymphal and adult ticks. However, we may have underestimated the efficiency of transstadial transmission in each tick developmental stage because a larval population fed on an infected mouse was used for transstadial transmission, and the larvae may have included SFTSV-infected and noninfected ticks. 

In this study, tick acquisition of SFTSV and transstadial transmission of SFTSV were demonstrated by RT-PCR detection of SFTSV RNA in ticks and confirmed by IFA detection of antibodies to SFTSV in mice fed on by SFTSV-infected nymphal and adult ticks. However, transovarial transmission of SFTSV in ticks was demonstrated only by RT-PCR, which may need further confirmation by isolation of SFTSV from tick eggs or larvae or from mice fed on by hatched larvae or by detection of antibodies to SFTSV in mice fed on by hatched larvae.

In conclusion, all developmental stages of *H. longicorn* ticks can acquire SFTSV by feeding on experimentally infected mice, and the ticks can transmit SFTSV to mice during feeding. The virus can be passed transstadially and transovarially in the developmental stages of the tick. However, the prevalence of SFTSV infection among ticks collected from vegetation is quite low, suggesting that ticks alone (at least *H. longicorn* ticks) may not be sufficient to maintain the virus in nature.
